# Quantitative Interpretation of Simulated Polymer Mean-Square Displacements

**DOI:** 10.3390/polym17040516

**Published:** 2025-02-17

**Authors:** George D. J. Phillies

**Affiliations:** Department of Physics, Worcester Polytechnic Institute, Worcester, MA 01690, USA; phillies@4liberty.net; Tel.: +1-508-754-1859

**Keywords:** polymer dynamics, mean-square displacement, polymer melt, polymer solution, computer simulation, scaling behavior, scaling exponents, power-law behavior

## Abstract

The time-dependencies of polymer mean-square displacements g(t) provide significant tests of some modern theories of polymer dynamics. Familiar models propose that g(t) is described by a series of power-law regimes $g\left(t\right)∼t^{\alpha }$, the models predicting values of α and time regimes within which those values will be found. g(t) has been obtained quantitatively over a wide range of times by means of computer simulations, permitting comparison of simulation measurements with these models. Here, we demonstrate a path for quantitatively analyzing g(t). We show that we can readily distinguish between regimes in which g(t) actually follows a power law in time, does not follow a power law in time, or has an inflection point. The method accurately determines local values of the exponent, without imposing any a priori assumption as to the exponent’s value.

## 1. Introduction

Many traditional models [1,2] of polymer solution dynamics predict that mean-square displacements g(t) of polymer molecules in solutions or in melts have the form of a power-law dependence,

(1) $$g\left(t\right)\equiv \langle {\left(\Delta x\left(t\right)\right)}^{2}\rangle =At^{\alpha },$$

where Δx(t) is a time-dependent displacement, 〈·〉 denotes an average, *t* is an elapsed time, α is an exponent, and *A* is a prefactor. Power-law dependencies are claimed to be valid over time intervals, with multiple power laws being separated by transition regimes serving to cover the full range of times. g(t) is the collective notation for a group of different displacements, notably $g_{1}\left(t\right)$, which describes the mean-square displacement of all the atoms in a polymer molecule, and $g_{3}\left(t\right)$, which describes the mean-square displacement of the polymer’s center of mass. The traditional models [1,2] predict the order in which different power-law regimes and exponents are encountered with increasing time, but do not, in general, predict the prefactor *A* or the time dependence of g(t) in transition regimes between neighboring power-law regions. To display g(t) as a function of time, recourse has uniformly been had to log–log plots, on which power laws such as that seen in Equation (1) appear as straight lines, each line’s slope being the corresponding exponent α.

Our objective in this proof-of-principle paper is to present a quantitative model-independent method for analyzing the time dependence of g(t). Our method does not assume that g(t) has a particular functional form, such as the power law of Equation (1). Contrast will be seen between our method and entirely legitimate studies that assumed that power-law behavior is present, so that segments of g(t) were fit to Equation (1) in order to obtain values for α.

Here, we invoke a general functional form that uniformly describes the time dependence of g(t) over the entirety of times for which g(t) has been obtained, without any assumption other than that the original data are consistent with a continuous line. Reported measurements appear to lie on smooth, monotonically increasing curves, so a reasonable choice of general form is a finite Taylor series expansion. The statistical spread in the data, in the reports we have examined, appears to be relatively independent of time on a log–log plot, so it is appropriate to take z=log(t) and write

(2) $$\log\left(g\left(t\right)\right)=\sum_{i=0}^{N}a_{i}z^{i},$$

for the series. Here, the $a_{i}$ are the fitting parameters, and *N* is the order of the fit. The $a_{i}$ are obtained by linear-least-squares. The scatter in measurements of log(g(t)) appears to be nearly independent of *t*, so issues concerning the statistical weights to be assigned to different points do not arise. As a cautionary note, Taylor series are used here as approximate interpolants covering the range over which measurements were made. It is not claimed that the series would be valid as an extrapolant beyond the range of *t* of the original measurements. What order of fit is appropriate? By increasing *N*, one reaches values of *N* for which the Taylor expansion agrees with the measurements, with further modest increases in *N* having no significant effect on the form of the fitted curve.

To clarify the behavior of g(t), we also determined its first and second logarithmic derivatives, with the first logarithmic derivative being

(3) $$K_{1}\equiv \frac{d\log(g(t))}{d\log(t)}=\sum_{i=1}^{N}ia_{i}z^{i-1}.$$

Note that $K_{1}$, being a logarithmic derivative, is dimensionless.

Our general search for papers on simulations of polymer dynamics revealed a considerable number of reports of g(t) for one or another definition of Δx(t). The reports are, uniformly, graphical. Measurements were digitized using Un-Scan-It 7.0, for the most part in manual mode [3]. Numerical analysis was made using Mathematica 12.1 [4].

This is a proof-of-principle paper. The objective is to demonstrate that our method works, not to discuss what it reveals about polymer physics. We selected three measurements of g(t) that serve to demonstrate significant aspects of our approach, namely Padding and Briels [5], with their Figure 1 showing mean-square atomic displacements, Brodeck et al. [6], with their Figure 1 showing the 400 K mean-square displacements, and Peng et al. [7], with their Figure 9a showing the mobility of their B beads. Why did we choose these three data sets? Analysis of the Padding and Briels results tests if the approach can usefully represent simple power-law behavior. One might be concerned as to how large an *N* is needed to represent a g(t) with multiple features, and what consequences would follow if the order of the fit was increased above the order needed to represent g(t) accurately. The effect of increasing the fit order is revealed by study of results from Brodeck et al. [6]. Finally, around an inflection point in g(t), a tangent line might mimic a power law. We inquire if actual power law regimes and inflection points can be distinguished, using results of Peng et al. [7].

It is legitimate to ask if the approach described here is generally applicable, or if the method leads to new physical results. A full-length paper answering these questions, based on close to a hundred sets of g(t) data as obtained by more than a dozen research groups, is now in preparation.

## 2. Tests of Finite Taylor Series as a General Functional Form

**Figure 1:**
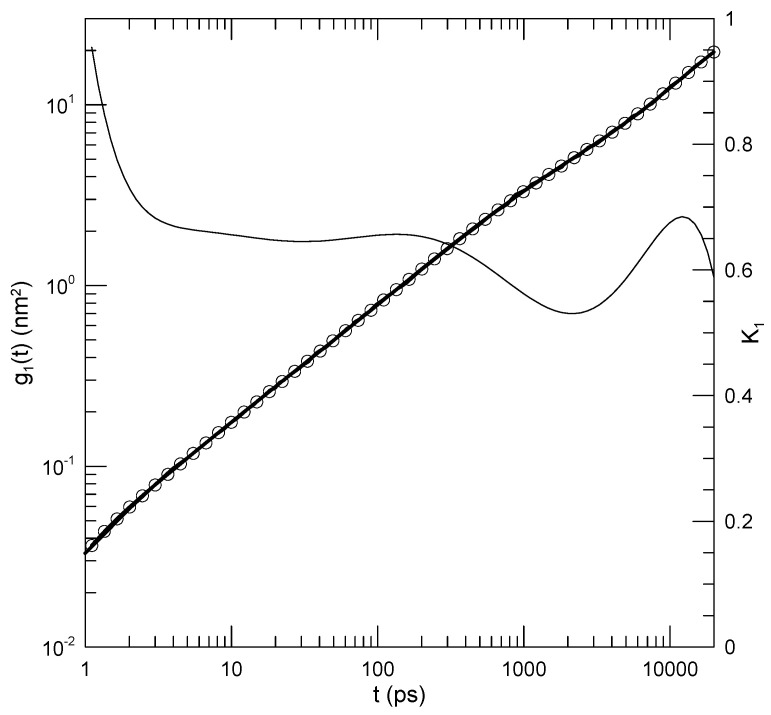
Mean-square atomic displacement $g_{1}\left(t\right)$ from a simulated polyethylene melt, results of Padding and Briels [5] (their Figure 1). The heavy solid line represents the digitized data from the original paper. Circles superposed on the heavy line are sampled points from an eighth-order polynomial fit to the data. The thin solid line is the first logarithmic derivative $K_{1}$ of the fitted polynomial. Between t=4 and t=130, $K_{1}=0.66\pm 0.01$, showing that there is power-law behavior in this region. At larger times, the slope $K_{1}$ decreases, with a minimum $K_{1}\approx 0.53$ near t=2100. $K_{1}$, being the logarithmic derivative of $g_{1}\left(t\right)$, is dimensionless.

In a series of papers, Padding and Briels [5,8,9,10] report simulations of a united-atom model for linear polyethylene, reporting, among other dynamic quantities, the diffusion coefficient, the shear relaxation modulus, the end-to-end vector’s time autocorrelation function, the single-chain coherent dynamic structure factor, and, of central interest here, various mean-square displacements. Figure 1 shows Padding and Briels’ determination of $g_{1}\left(t\right)$, the mean-square displacement of their individual united atoms. We fit $\log(g_{1}\left(t\right))$ to an eighth-order polynomial in log(t). One sees in Figure 1 that the agreement between our polynomial fit (circles) and the original measurements (heavy line) is excellent.

The original authors report that $g_{1}\left(t\right)$ can be described by two power laws, one with α=0.65 for t<200 ps and another with α=0.57 for t>200 ps. On a log–log plot, a power law would appear as a straight line whose slope equals α. Our results are seen in Figure 1. As seen in the figure, for times of a few picoseconds up to 200 ps, the calculated slope $K_{1}$ is very nearly constant, corresponding to α≈0.66±0.01 in agreement with Padding and Briels. For t>200 ps, our polynomial fit reveals that the slope decreases very slightly, to perhaps 0.53 or so, and then increases again back toward 0.65. This gentle modulation of the slope is revealed by our fitting process, but would have been masked a fit to an assumed power-law behavior. A simple power law would be approximately consistent with these measurements, as seen in Padding and Briels’ Figure 1, with the deviations of the data from a simple power law being modest.

Our fitting process is thus seen to reveal power-law behavior when such is present.

Brodeck et al. [6] report atomistic molecular dynamics simulations of a polyethylene oxide-polymethylmethacrylate mixture at four temperatures. Their interest was the dynamic asymmetry between the two components, with polymethylmethacrylate density fluctuations relaxing much more slowly than polyethylene oxide density fluctuations. They report an analysis using Rouse mode decomposition, mean-square displacements, non-Gaussian parameters for the distribution of mean-square displacements, and comparison with a simple bead-spring model.

**Figure 2:**
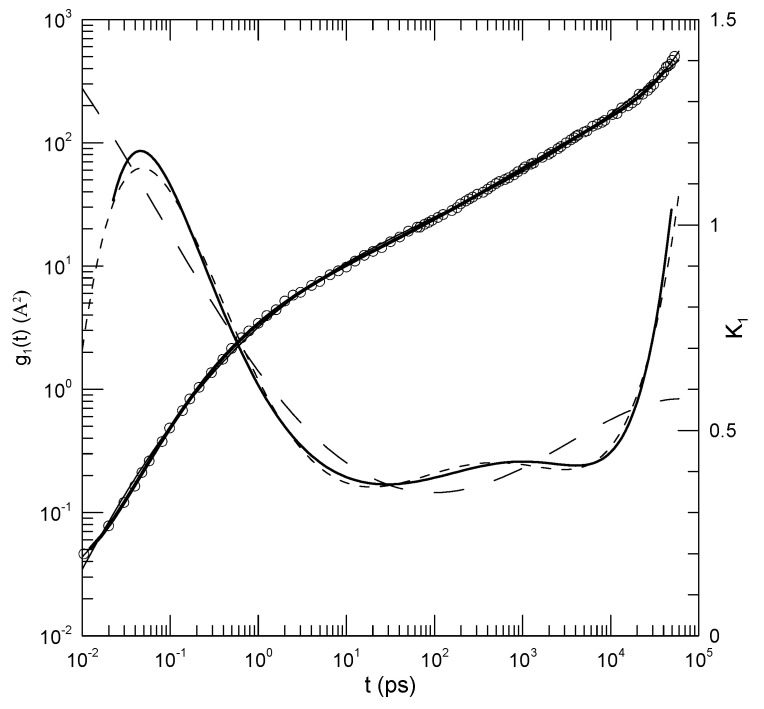
Determinations of the mean-square displacement from results of Brodeck et al. [6] at 400 K. Circles are digitized results from the original paper. The three solid lines through the points (indistinguishable over most of the graph) represent fits to eighth, sixth, and fifth order polynomials. The three lines not passing through the points are the first logarithmic derivative $K_{1}$ from the eighth-order (solid line), sixth-order (short dashes), and fifth-order (long dashes) fits to the original measurements. The first derivatives from the eighth- and sixth-order fits are nearly the same. Note that $K_{1}$, like the exponent α of $t^{\alpha }$, is a dimensionless pure number.

Figure 2 shows their calculated mean-square displacements for their blend at 400 K. Fits to fifth-, sixth-, and eighth-order polynomials lead to the solid lines that pass very nearly uniformly through the data points. The lines are almost completely overlapping, except noting the upper right. The computed first logarithmic derivative $K_{1}$ corresponds to the thin solid and dashed lines. Over times 1.2 × $10^{1}–4.9$ × $10^{4}$, the first derivative from the eighth-order fit (solid line) increases from approximately 0.37 to 1.03. At the lower extremum, the slope becomes considerably larger, reaching ≈1.2 at short times. At times t>1 × $10^{4}$, we find that $\log(g_{1}\left(t\right))$ follows a smooth curve having a continuously increasing slope, with, as reported by Brodeck et al., a tangent having slope ≈ 1 at time ≈ 4 × $10^{4}$.

The fitting process is thus seen to describe time dependencies that are more complex than single power laws.

Peng et al. [7] report simulations of a united-atom model of flexible polymers having bead-bead Lennard-Jones interactions, beads of each chain being linked with a finitely extensible nonlinear-elastic potential. To these were added chains made more rigid by giving them significant bond-bending and torsional potentials. The blends were of interest because they contained two polymer species with very different glass temperatures and mobilities.

**Figure 3:**
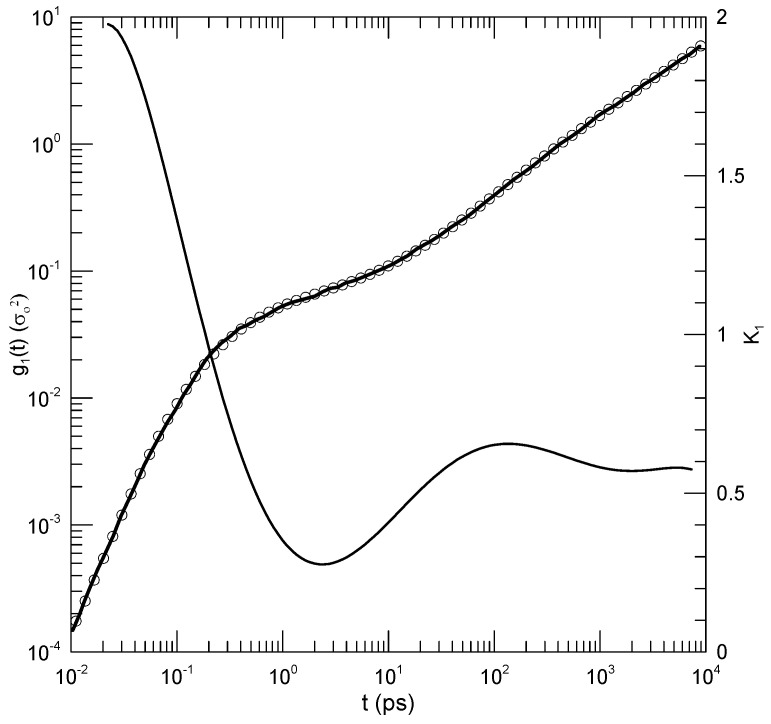
Mean-square atomic displacement $g_{1}\left(t\right)$ from Peng et al. [7]. The original data and an eighth-order polynomial fit are represented by a solid line (the data) and circles superposed on the line (sampled points from the polynomial fit). The other solid line, with deep minimum near t=2.5, is the first logarithmic derivative $K_{1}$ of the fitted function. The first derivative curve shows that the possible power law region near t=2.5 is actually an inflection point, while the region having t≥1000 is indeed a power-law regime with $K_{1}\equiv \alpha \approx 0.58$.

Figure 3 shows mean-square displacements of beads of the non-rigid polymers in a mixture to which a small amount of the more rigid polymer has been added (Peng et al.’s Figure 9a, $N_{A}=5$). The solid line represents an eighth-order polynomial fit, which describes $\log(g_{1}\left(t\right))$ well at almost all times. We see for long times (t>100) that $\log(g_{1}\left(t\right))$ increases nearly linearly in time, with a slope $d\log(g_{1}\left(t\right))/d\log\left(t\right)\approx 0.58$. At short times (t≤0.1), $g_{1}\left(t\right)$ increases rapidly with increasing time, with a slope approaching 2. Of particular interest for this paper is the behavior of $\log(g_{1}\left(t\right))$ for times near t=2.5. In this range, $\log(g_{1}\left(t\right))$ superficially appears to increase linearly with increasing time, implying power-law behavior $g_{1}\left(t\right)∼t^{\alpha }$. However, $K_{1}$, the first derivative, simply has a minimum at t=2.5, the slope having a parabolic dependence on log(t) around this point. The region near t=2.5 is thus revealed to be an inflection point of $\log(g_{1}\left(t\right))$, not a local power-law regime.

## 3. Discussion

Here, we have demonstrated an approach to analyzing simulated measurements of the mean-square displacement g(t) of polymer molecules. The approach is of interest because there are theoretical models that predict that g(t) is described by a series of power law regimes in *t*, the power-law regimes being separated by transition regimes. Power law regimes are revealed as regions of *t* in which the logarithmic derivative of g(t) is nearly constant. As shown above, our approach finds such regions when they exist. The same method also identifies regions whose time dependence is not a power law, and distinguishes between a power law regime and an inflection point in log(g(t)).

This paper represents a proof-of-principle test. It is appropriate to ask if the approach described here is generally applicable, as opposed to the above discussion reflecting the behavior of a few outlying results. It is also appropriate to ask if the approach leads to new physically interesting results. A full length paper, answering this question by analyzing close to a hundred sets of g(t) data, is now in preparation.

## Data Availability

Data are contained within the article.
